# Synthetic study toward the diterpenoid aberrarone

**DOI:** 10.3762/bjoc.18.173

**Published:** 2022-11-30

**Authors:** Liang Shi, Zhiyu Gao, Yiqing Li, Yuanhao Dai, Yu Liu, Lili Shi, Hong-Dong Hao

**Affiliations:** 1 Department Shaanxi Key Laboratory of Natural Products & Chemical Biology, College of Chemistry & Pharmacy, Northwest A&F University, Yangling, Shaanxi 712100, China; 2 State Key Laboratory of Chemical Oncogenomics, Guangdong Provincial Key Laboratory of Chemical Genomics, Peking University Shenzhen Graduate School, Shenzhen, Guangdong 518055, China

**Keywords:** aberrarone, C–H insertion, gold, Pauson–Khand, total synthesis

## Abstract

An approach to aberrarone, an antimalarial diterpenoid natural product with tetracyclic skeleton is reported. Key to the stereoselective preparation of the 6-5-5 tricyclic skeleton includes the mediation of Nagata reagent for constructing the C1 all-carbon quaternary centers and gold-catalyzed cyclopentenone synthesis through C–H insertion.

## Introduction

**Figure 1:**
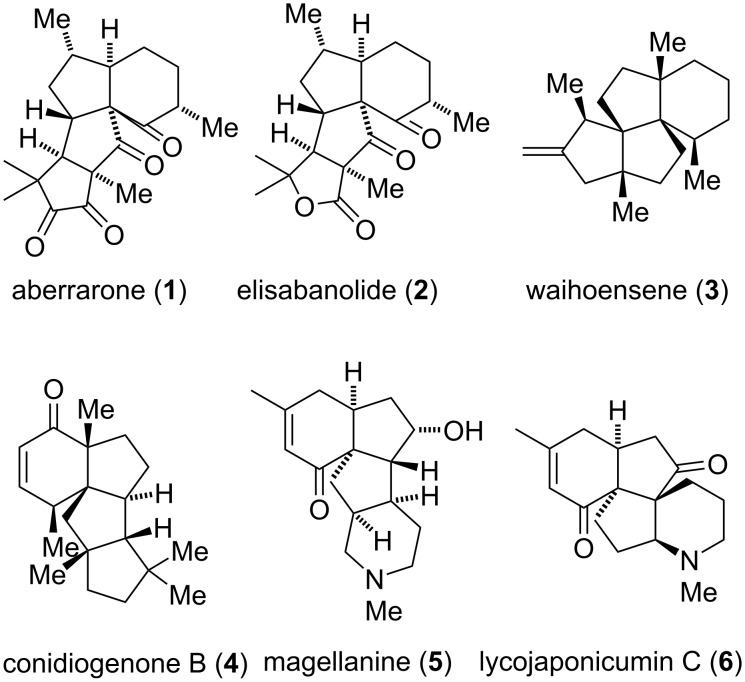
Selected representative natural products with 6-5-5 tricyclic skeleton.

Marine natural products have found myriad use in new drug development, exemplified by ET-743 and eribulin [1]. Back in 1990s, Rodriguez and co-workers isolated a rich array of terpenoid natural products from the Caribbean sea whip, *Pseudopterogorgia elisabethae* with unprecedented carbon skeleton, most of which showed antitumor, antituberculosis and antimalarial activities [2–6]. Among these structurally intriguing natural products, aberrarone (**1**) shows antimalarial activity against the chloroquine-resistant strain of *Plasmodium falciparum* (IC_50_ = 10 μg/mL) [7]. Structurally, aberrarone possesses an unusual tetracyclic carbon skeleton yet-to-be found in *Pseudopterogorgia elisabethae* species, although related cyclohexane-angularly-fused triquinane systems have been found in waihoensene (**3**), conidiogenone (**4**), lycopodium alkaloids magellamine (**5**) and lycojaponicumin C (**6**) (Figure 1). Its seven stereogenic centers, including two all-carbon quaternary centers, together with the non-enolizable cyclic α-diketone moiety collectively render aberrarone as an attractive but challenging synthetic target. Its congener elisabanolide (**2**) with a lactone in the D ring shows their potential biosynthetic relationship [2]. These natural products have been popular synthetic targets mainly due to their intriguing structural features. For example, several total syntheses of **3**–**6** have been reported [8–29]. Previously, two synthetic studies of aberrarone were reported [30–31] and more recently, Carreira and co-workers reported [32] the first total synthesis of aberrarone through an impressive cascade reaction including a gold-catalyzed Nazarov cyclization, a cyclopropanation followed by intramolecular aldol reaction to forge the A, B and D rings. Impressed by the structural features and biological profiles, our group embarked a project on the total synthesis of this natural product. Herein, we report our stereoselective synthesis of its 6-5-5 tricyclic skeleton.

**Scheme 1:**
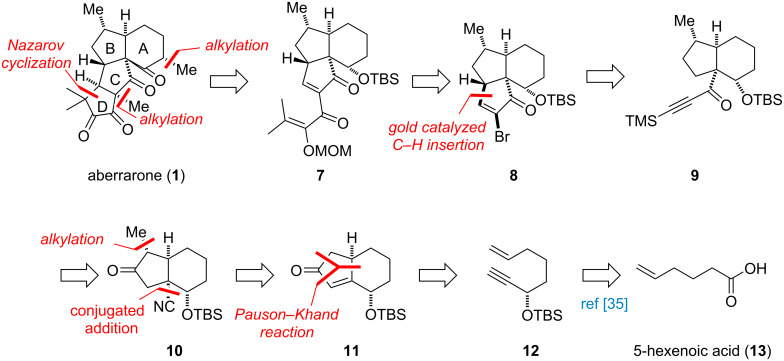
Retrosynthetic analysis of aberrarone (**1**).

Our retrosynthetic analysis is shown in Scheme 1. For the formation of the D ring with one quaternary carbon stereocenter and 1,2-dikeone moiety, Nazarov cyclization [33] of **7** was proposed for synthesizing this challenging moiety. The corresponding precursor cyclopentenone **8** may be prepared from alkynone **9** through a gold-catalyzed C–H insertion [34]. Alkynone **9** could be achieved through functional transformation from **10**, which itself would be prepared through methylation and conjugate addition from Pauson–Khand adduct **11**. This cyclopentenone could be readily accessed from 1,7-enyne **12** which could be obtained through the reported procedure [35] from the commercially available 5-hexenoic acid.

## Results and Discussion

**Scheme 2:**
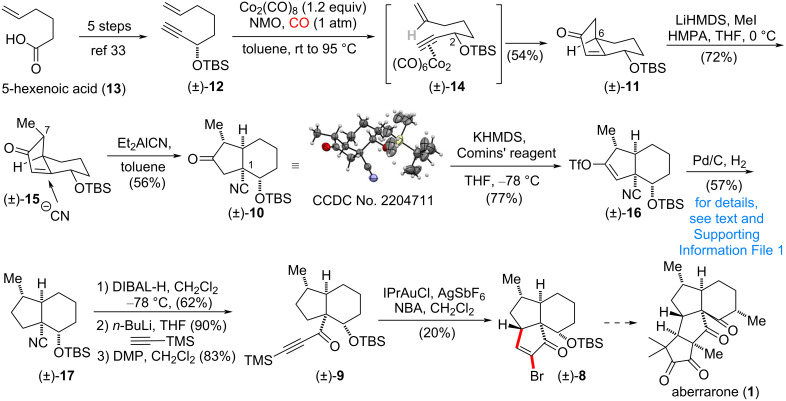
Synthetic study toward aberrarone (**1**).

Our synthetic route commenced from known compound **12** which is readily accessed from 5-hexenoic acid through a reported procedure [35]. In the mediation of Co_2_(CO)_8_, the 6-5 bicyclic skeleton [36] was constructed with the right configuration at C6, and the explanation of this stereoselectivity is possible through the conformation of **14** where the OTBS group is in pseudoequatorial position (Scheme 2). Therefore, the Pauson–Khand reaction proceeded to afford **11** containing an α-H at C6. From this intermediate, to our delight, the stereoselective attachment of the requisite methyl group through the corresponding lithium enolate occurred from the convex face of the bicyclic ring system [37]. After these two continuous stereocenters were successfully installed, the expected challenging all-carbon quaternary center at C1 was constructed utilizing the Nagata reagent (Et_2_AlCN). By using this strategy, the stereogenic center at C1 was synthesized, along with a smooth attachment of the cyanate group served for further functional group transformation to construct the C ring through C–H insertion. The stereochemistry finding of this conjugate addition from the convex face of the 6-5 ring system was further confirmed through X-ray crystallographic analysis.

With the key intermediate **10** in hand, we were in a position to test the planned two-step transformation including the palladium-catalyzed reductive cross coupling with HCO_2_H followed by Pd/C-catalyzed hydrogenation. To our surprise, the hydrogenation turned out to be a difficult transformation due to the steric hindered environment of the trisubstituted double bond, mainly caused by the bulky OTBS group. However, direct subjection of compound **16** to hydrogenation [38] afforded reduction of both triflate and double bond. The plausible pathway for this facile transformation might proceed with first hydrogenation followed by the substitution of the labile triflate ester (for details, see Supporting Information File 1). Moving forward, compound **17** was further converted into alkynone **9** through DIBAL-H reduction, nucleophilic addition and Dess–Martin oxidation. At this stage, the pivotal C–H insertion step was tried under the reported conditions [34], and cyclopentenone **8** was successfully obtained. Further study with cross coupling or halogen–magnesium exchange shows this moiety is inert for functional group transformation. The attempt for constructing the D ring is currently undergoing.

## Conclusion

In summary, we have developed an approach to assemble the tricyclic skeleton of aberrarone through stereoselective methylation, conjugate addition and gold-catalyzed C–H insertion from the readily accessed cyclopentenone. Further work to access natural product aberrarone from the key intermediate cyclopentenone **8** is currently underway, and will be reported in due course.

## Supporting Information

The crystallographic data of compound **10** (CCDC 2204711) has been deposited at the Cambridge Crystallographic Database Center (http://www.ccdc.cam.ac.uk).

File 1 Characterization data and ^1^H NMR, ^13^C NMR, and HRMS spectra of the compounds.
