# Supplementary material for: Synthetic study toward the diterpenoid aberrarone
Source: Beilstein J Org Chem. 2022 Nov 30;18:1625–8. doi: 10.3762/bjoc.18.173 (PMC9727272; doi:10.3762/bjoc.18.173)
Supplement: File 1 — Characterization data and 1H NMR, 13C NMR, and HRMS spectra of the compounds. [file Beilstein_J_Org_Chem-18-1625-s001.pdf]

BEILSTEIN JOURNAL OF ORGANIC CHEMISTRY

Supporting Information

for

Synthetic study toward the diterpenoid aberrarone

Liang Shi, Zhiyu Gao, Yiqing Li, Yuanhao Dai, Yu Liu, Lili Shi and Hong-Dong Hao

Beilstein J. Org. Chem. **2022,** *18,* 1625–1628. doi:10.3762/bjoc.18.173

Characterization data and ^{1}H NMR, ^{13}C NMR, and HRMS spectra of the compounds

License and Terms: This is a supporting information file under the terms of the Creative Commons Attribution License (https://creativecommons.org/licenses/by/4.0). Please note that the reuse, redistribution and reproduction in particular requires that the author(s) and source are credited and that individual graphics may be subject to special legal provisions.
The license is subject to the *Beilstein Journal of Organic Chemistry* terms and conditions: (https://www.beilstein-journals.org/bjoc/terms)

Table of contents

1. General experimental details……S2

 1.1. Materials……S2

 1.2. Instrumentation……...S3

2. Experimental procedures……...S3

3. References……...S9

4. ^{1}H and ^{13}C NMR spectra……S10

1. General experimental details

Unless otherwise stated, all reactions were performed with magnetic stirring under a positive pressure of nitrogen or argon gas. Over-dried glassware (over temperature of 150 ºC) was further dried with a heat-gun at 650 ºC under vacuum, followed by back-filling with inert gas, three times and fitted with rubber septa prior to use. Solids were added under inert gas counter flow or were dissolved and transferred in the appropriate solvent. Solutions and liquids reagents were transferred to reaction vessels by oven-dried stainless-steel cannulas or nitrogen flushed syringes. Low temperature reactions were carried out in a Dewar vessel filled with acetone/dry ice (–78 °C) or distilled water/ice (0 °C). High temperature reactions were conducted using a heated silicon oil bath in reaction vessels equipped with a reflux condenser.

1.1 Materials

Dry Tetrahydrofuran (THF), Dichloromethane (CH_2Cl_2), ethanol (EtOH), toluene (PhMe) and methanol (MeOH) were purchased from Tansoole company and stored over molecular sieves. Ethyl acetate (EtOAc), petroleum ether (PE), methyl *tert*-butyl ether (*t*-BuOMe) used specifically for extraction and flash column chromatography were purchased from commercial sources. All other solvents and regents were also purchased from commercial sources (Sigma Aldrich, Energy chemical, 3A, Adamasect.).

Reactions were monitored by thin-layer chromatography (TLC) using silica gel F254 pre-coated glass plates (Merck) and visualized by exposure to ultraviolet light (λ = 254 nm) or by staining with aqueous potassium permanganate ($KMnO_4$) solution (7.5 g $KMnO_4$, 50 g K_2CO_3, 6.25 mL aqueous 10% NaOH, 1000 mL distilled H_2O), phosphomolybdic acid hydrate (PMA) solution (10.0 g PMA, 100 mL EtOH) followed by heating with a heat gun (150–600 °C). Flash column chromatography was performed using silica gel (60 Å, 40–63 μm, Merck) and a forced flow of eluent.

1.2 Instrumentation

NMR spectra were recorded on a Brucker Avance III HD 500 MHz and 400 MHz spectrometer equipped with a CroProbe TM. Chemical shifts were reported in parts per million (ppm) respectively to the residual solvent signal (^{1}H NMR: $CDCl_3$: 7.26 ppm; C_6D_6: 7.16 ppm; ^{13}C NMR: $CDCl_3$ 77.2 ppm; C_6D_6: 128.06 ppm). The reported data is represented as follows: chemical shift

in parts per million (ppm, δ scale), multiplicity, coupling constants *J* in Hz, integration intensity and proton assignment. Abbreviations used for analysis of multiplets are as follows: s (singlet), br (broad singlet), d (doublet), t (triplet), q (quartet), quin (quintet), h (hextet), and m (multiplet). Variable temperature NMR spectroscopy was performed at the Northwest A&F University NMR facility.

Mass spectroscopy (MS) experiments were performed in high resolution with an AB SCIEX Triple TOF 5600+ spectrometer (AB SCIEX, Boston, MA, USA). IR spectra were recorded on a Perkin-Elmer Frontier FT-IR spectrometer.

Single-crystal X-ray diffraction data was collected on a Bruker D8-Quest diffractometer equipped with a photon 100 detector by using a graphite monochromator utilizing Mo-Kα radiation (λ = 0.71073 Å). Data integration and absorption correction were processed by the SAINT and SADABS programs. The structures were solved by intrinsic phasing with the SHELXS and refined by full-matrix least-squares methods on F2 by using the SHELXL-2018 program. The hydrogen atoms on C atoms were fixed at the calculated positions and refined by a riding model, with Uiso(H) = 1.2 Ueq(C).

2. Experimental procedures

Hydrogenation was tried on compounds S1 and S2. To our surprise, hydrogenation of S1 failed in our hand.

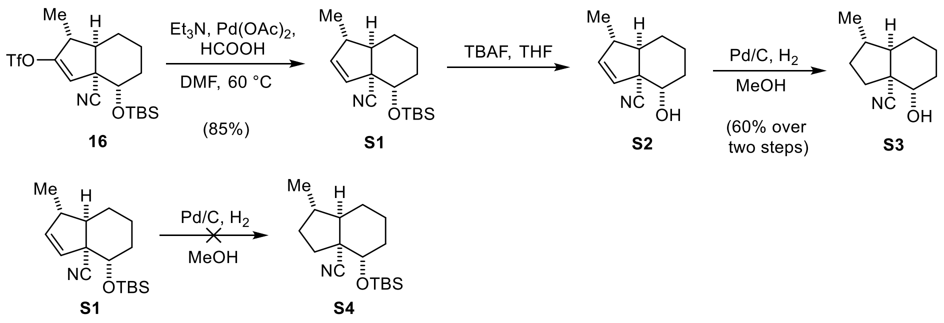

Construction the C ring through radical cyclization or [3 + 2] cycloaddition was also tried but failed.

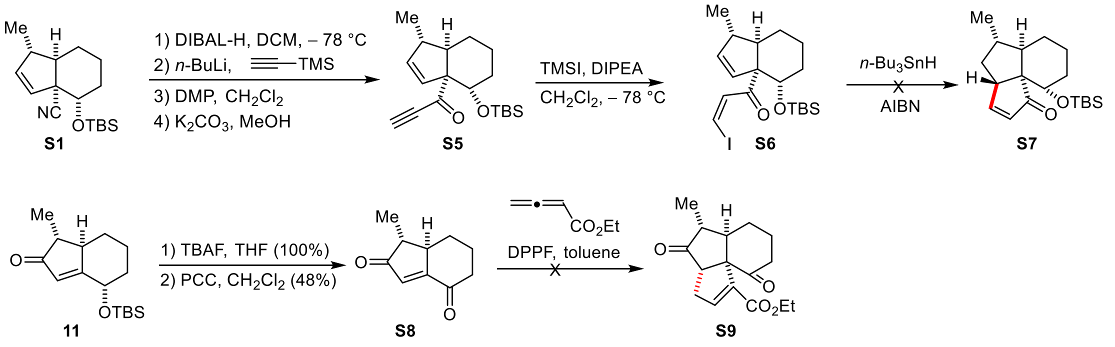

Synthesis of compound 11

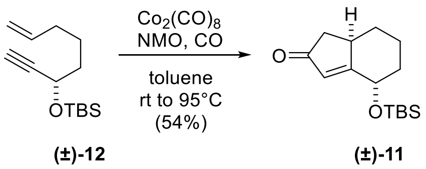

To a stirred solution of **12**[1] (635 mg, 2.66 mmol) in dry toluene (20 mL) at rt was added $Co_2(CO)_8$ (999 mg, 2.93 mmol) under 1 atm of CO, the mixture was stirred at rt for 1 h. After the complete transformation of the starting material, the reaction mixture was added NMO (1.563 g, 13.3 mmol) and heated to 95 °C overnight. The reaction mixture was cooled to room temperature, and concentrated under reduced pressure, the residue was purified by flash column chromatography (EtOAc/petroleum ether = 1:25) to give product **11** (380 mg, 1.43 mmol, 54%) as a yellow oil.

Data for 11: R_f = 0.28 (silica, EtOAc/petroleum ether = 1:20, stains with PMA); **^{1}H NMR (500 MHz, chloroform-*d*):** δ 5.82 (s, 1H), 4.73 (s, 1H), 3.10 – 3.02 (m, 1H), 2.57 (dd, J = 19.1, 6.2 Hz, 1H), 2.19 – 2.13 (m, 1H), 1.99 – 1.93 (m, 3H), 1.60 – 1.45 (m, 3H), 1.06 (q, J = 25.4, 12.7 Hz, 1H), 0.88 (s, 9H), 0.07 (s, 3H), 0.00 (s, 3H) ppm; **^{13}C NMR (125 MHz, chloroform-*d*)**: δ 209.7, 184.1, 125.8, 66.9, 42.4, 37.7, 35.7, 35.6, 25.8, 19.3, 18.2, –4.6, –4.9 ppm; **HRMS ESI** calcd for $C_{15}H_{26}O_2SiNa^+$ $[M+Na]^+$ 289.1594, found 289.1594; **IR (neat)** ν_{max} 2940, 2861, 1705, 1709, 1255, 1020, 839, 781 cm^{-1}.

Synthesis of compound 15

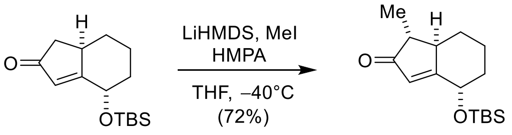

11 15

To a solution of the compound **11** (283 mg, 1.06 mmol) in tetrahydrofuran (10 mL) was added LiHMDS (1.27 ml, 1.27 mmol, 1.0 M in THF) at −40 °C. After stirred for 1 h, HMPA (0.35 mL, 1.27 mmol) and MeI (0.08 mL, 1.27 mmol) was added to the reaction mixture. The reaction was quenched by saturated NH_4Cl aqueous solution and extracted with EtOAc (3 × 10 mL). The combined organic extract was dried over anhydrous Na_2SO_4 and concentrated in vacuo. The crude product was purified via flash chromatography (EtOAc/petroleum ether = 1:30) to provide **15** (214 mg, 0.763 mmol, 72%) as a yellow oil.

Data for 15: R_f = 0.37 (silica, EtOAc/petroleum ether = 1:20, stains with PMA); **^{1}H NMR (500 MHz, chloroform-*d*):** δ 5.75 (s, 1H), 4.71 – 4.66 (m, 1H), 2.63 – 2.52 (m, 1H), 2.21 – 2.11 (m, 1H), 1.95 – 1.83 (m, 3H), 1.55 – 1.40 (m, 2H), 1.12 (d, J = 7.7 Hz, 3H), 1.06 – 0.98(m, 1H), 0.84 (s, 9H), 0.03 (s, 3H), −0.05 (s, 3H) ppm; **^{13}C NMR (125 MHz, chloroform-*d*)**: δ 211.7, 181.6, 124.8, 66.8, 48.0, 46.5, 35.3, 34.6, 25.7, 19.3, 18.1, 15.2, −4.7, −4.9 ppm; **HRMS ESI** calcd for $C_{16}H_{28}O_2SiNa^+$ $[M+Na]^+$ 303.1751, found 303.1747; **IR (neat)** ν_{max} 2934, 2859, 1711, 1254, 1165, 1078, 837, 779 cm^{-1}.

Synthesis of compound 10

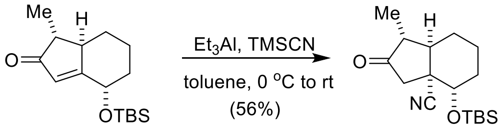

15 10

Preparation of Nagata reagent: To a solution of Et_3Al (1.0 mL, 1.0 mmol, 1.0 M in toluene) was added TMSCN (125 µL, 1.0 mmol) at rt Then the solution was heated to 110 °C. After 1 h, the formed Nagata reagent was cooled down to rt and used for next step.

To a stirred solution of **15** (50.6 mg, 0.18 mmol) in dry toluene (2 mL) at 0 °C was added Et_2AlCN (0.9 mL, 0.9 mmol), after 2 h, the reaction mixture was quenched by saturated $NaHCO_3$ aqueous solution (2 mL) and extract with EtOAc (3 × 2 mL). The combined organic layers were washed with brine, concentrated and purified by flash chromatography (EtOAc/petroleum ether =

1:25) to afford product **10** (31 mg, 0.10 mmol, 56%) as a white solid.

Data for 10: ***R*f** = 0.50 (silica, EtOAc/petroleum ether = 1:8, stains with PMA); **^{1}H NMR (500 MHz, chloroform-*d*):** δ 3.38 (dd, J_1 = 11.4, 4.0 Hz, 1H), 2.88 – 2.81 (m, 1H), 2.63 – 2.56 (m, 1H), 2.39 – 2.33 (m, 1H), 2.18 – 2.10 (m, 1H), 1.94 – 1.87 (m, 2H), 1.79 – 1.68 (m, 3H), 1.47 – 1.39 (m, 1H), 1.10 (d, *J* = 6.9 Hz, 3H), 0.91 (s, 9H), 0.08 (s, 3H), 0.05 (s, 3H) ppm; **^{13}C NMR (125 MHz, chloroform-*d*)**: δ 214.7, 121.1, 71.4, 49.3, 46.9, 45.4, 43.5, 32.7, 25.8, 22.3, 19.5, 18.1, 13.5, –3.6, –4.7 ppm; **HRMS ESI** calcd for $C_{17}H_{29}NO_2SiNa^+$ $[M+Na]^+$ 330.1860, found 330.1856; **IR (neat)** ν_{max} 2935, 2861, 2239, 1747, 1466, 1257, 1116, 838, 778 cm^{-1}.

Synthesis of compound 16

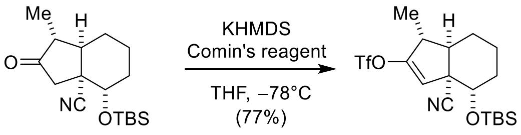

10 **16**

A solution of **10** (593 mg, 1.93 mmol) in dry tetrahydrofuran (20 mL) at –78 °C was added dropwise KHMDS (2.9 mL, 2.9 mmol, 1.0 M solution in THF). After 40 min, a solution of Comin's reagent (1.137 g, 2.9 mmol) in dry tetrahydrofuran (10 mL) was added. The reaction mixture was stirred at –78 °C for 2 h. Then the reaction mixture was quenched with saturated aqueous NH_4Cl, extracted with *t*-BuOMe (3 × 20 mL), dried over anhydrous Na_2SO_4 and concentrated in vacuo. The residue was purified by flash chromatography (EtOAc/petroleum ether = 1:60) to give compound **16** (652 mg, 1.49 mmol, 77%) as a yellow oil. Compound **16** was used immediately due to the instability.

Synthesis of compound 17

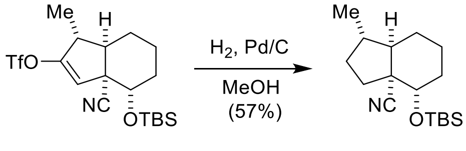

16 **17**

To a solution of the compound **16** (62 mg, 0.142 mmol) in dry methanol (4 mL) were added 10% Pd/C (10 mg). The mixture was hydrogenated with a H_2 balloon until the starting material disappeared on TLC. The reaction mixture was filtered through a short silica gel column to

remove the catalyst. Evaporation of the solvent and purification of the residue over a flash column chromatography (EtOAc/petroleum ether = 1:40) to give product **17** (24 mg, 0.08 mmol, 57%) as a colorless oil.

Data for 17: $\boldsymbol{R}_f$ = 0.61 (silica, EtOAc/petroleum ether = 1:20, stains with PMA); **^{1}H NMR (400 MHz, benzene-*d*$_6$)** δ 3.17 (dd, J_1 = 10.6, J_1 = 5.0, 1H), 2.07 – 1.99 (m, 1H), 1.85 – 1.76 (m, 1H), 1.65 – 1.51 (m, 5H), 1.47 – 1.32 (m, 2H), 1.28 – 1.21 (m, 2H), 1.01 (s, 9H), 0.99 – 0.96 (m, 1H), 0.63 (d, J = 6.4, 3H), 0.03 (s, 3H), 0.00 (s, 3H) ppm; **^{13}C NMR (100 MHz, benzene-*d*$_6$)** δ 122.5, 71.1, 53.1, 50.4, 33.8, 33.2, 32.9, 30.6, 26.0, 22.2, 20.0, 19.5, 18.3, −3.6, −4.8 ppm; **HRMS ESI** calcd for $C_{17}H_{31}NOSiNa^+$ $[M+Na]^+$ 316.2067, found 316.2066.

Synthesis of compound S10

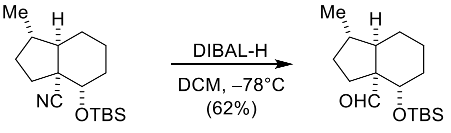

17 **S10**

To a solution of alcohol **17** (162 mg, 0.55 mmol) in dry dichloromethane (8 mL) was added DIBAL-H (0.83 mL, 0.83 mmol, 1.0 M in hexanes) at –78 °C under argon. After completing the reaction, the mixture was quenched by MeOH (1 mL) and then saturated Rochelle's salt solution (10 mL) was added, and the mixture was stirred vigorously for 2 h at room temperature until the two layers were clear and the aqueous phase was extracted with *t*-BuOMe (3 × 10 mL). The combined organic layers was washed with brine, dried over anhydrous Na_2SO_4 and concentrated under reduced pressure. Afterwards, the residue was purified through flash column chromatography (EtOAc/petroleum ether = 1:8) to yield **S10** (100 mg, 0.34 mmol, 62%) as a colorless oil. Aldehyde **S10** was directly used for next step.

Synthesis of compound S11

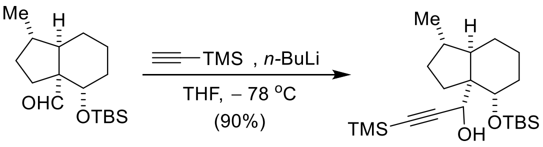

S10 **S11**

To a stirred solution of trimethylsilylacetylene (0.4 mL, 2.82 mmol) in tetrahydrofuran (6 mL) at –78 °C was added *n*-BuLi (1.73 mL, 2.77 mmol, 1.6 M in hexanes), the reaction mixture was stirred for 60 min and aldehyde **S10** (162 mg, 2.82 mmol) in tetrahydrofuran (5 mL) was added

dropwise. After 1 h at –78 °C, the reaction mixture was quenched by saturated aqueous NH_4Cl solution (10 mL) and the aqueous layer was extracted with *t*-BuOMe (3 × 10 mL). The combined organic layers were washed with brine (20 mL), dried by Na_2SO_4. The solvent was removed by rotary evaporation to give crude product, which was purified through flash column chromatography (EtOAc/petroleum ether = 1:80) to yield **S11** (198 mg, 0.5 mmol, 90%) as a light yellow oil. Compound **S11** was obtained as a single diastereomer and directly used for the next step.

Synthesis of compound 9

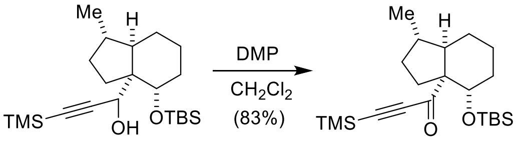

S11 9

To a stirred solution of **S11** (51 mg, 0.13 mmol) in CH_2Cl_2 (3 mL) were added Dess–Martin periodinane (92 mg, 0.2 mmol) at room temperature. After 1 h, the reaction mixture was quenched with saturated aqueous Na_2SO_3 and extracted with *t*-BuOMe (3 × 5 mL). The combined organic layer was washed with brine, dried over anhydrous Na_2SO_4 and concentrated in vacuo. The residue was purified by flash column chromatography (EtOAc/petroleum ether = 1:100) to produce **9** (42 mg, 0.11 mmol, 83%) as a light yellow oil.

Data for 9: $\boldsymbol{R}_f$ = 0.95 (silica, EtOAc/petroleum ether = 1:30, stains with PMA); **^{1}H NMR (500 MHz, benzene-*d*$_6$):** δ 3.76 (dd, J_1 = 10.0 Hz, J_2 = 3.8 Hz, 1H), 2.61 – 2.54 (m, 1H), 2.09 – 2.04 (m, 2H), 1.87 – 1.81 (m, 1H), 1.79 – 1.73 (m, 1H), 1.65 – 1.54 (m, 3H), 1.36 – 1.28 (m, 2H), 1.27 – 1.23 (m, 1H), 1.15 – 1.10 (m, 1H), 1.02 (s, 9H), 0.83 (d, J = 6.7 Hz, 3H), 0.12 (s, 3H), 0.10 (s, 9H), 0.06 (s, 3H) ppm; **^{13}C NMR (125 MHz, benzene-*d*$_6$)**: δ 191.0, 128.3, 104.2, 96.2, 74.9, 63.3, 49.4, 36.9, 33.6, 31.6, 31.3, 26.2, 24.2, 20.0, 18.5, –0.7, –3.7, –4.8 ppm; **HRMS ESI** calcd for $C_{22}H_{40}O_2Si_2Na^+$ $[M+Na]^+$ 415.2459, found 415.2459; **IR (neat)** ν_{max} 2952, 2866,1667, 1422, 1254, 1216, 1137, 1092, 848 cm^{-1}.

Synthesis of compound 8

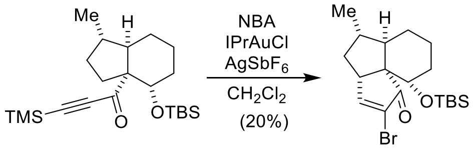

NBA (12.5 mg, 0.09 mmol), IPrAuCl (1.9 mg, 0.003 mmol), and $AgSbF_6$ (2.1 mg, 0.006 mmol) were added in this order to a mixture of ynone **9** (23.3 mg, 0.06 mmol) in dichloromethane (2 mL) at room temperature. The reaction mixture was stirred at room temperature for 10 h. Upon completion, the mixture was concentrated and the residue was purified by chromatography on silica gel (EtOAc/petroleum ether = 1:80) to afford the desired product **8** (5 mg, 0.012 mmol, 20%) as a colorless oil.

Data for 8: $\boldsymbol{R}_f$ = 0.62 (silica, EtOAc/petroleum ether = 1:30, stains with PMA); **^{1}H NMR (400 MHz, benzene-*d$_6$*):** δ 6.94 (d, J = 2.84 Hz ,1H), 3.55 (dd, J = 11.9, 4.7 Hz, 1H), 2.76 (td, J = 5.5, 2.9 Hz, 1H), 2.45 (qd, J = 12.4, 3.4 Hz, 1H), 1.80 – 1.74 (m, 1H), 1.65 – 1.60 (m, 2H), 1.47 (dt, J_1 = 13.6, J_2 = 3.6 Hz, 2H), 1.34 – 1.29 (m, 2H), 1.06 – 1.01 (m, 2H), 0.9 (s, 9H), 0.58 (d, J = 2.8 Hz, 3H), 0.02 (s, 3H), −0.03 (s, 3H) ppm; **^{13}C NMR (100 MHz, benzene-*d$_6$*):** δ =201.0, 161.3, 123.6, 75.2, 60.3, 51.3, 48.0, 39.1, 37.7, 31.5, 25.9, 21.7, 20.6, 18.2, 17.4, −3.3, −4.9 ppm; **HRMS ESI** calcd for $C_{19}H_{31}BrO_2SiNa^+$ $[M+Na]^+$ 421.1169, found 421.1170. **IR (neat)** ν_{max} 2926, 2856, 1716, 1257, 1103, 841, 815 cm^{-1}.

3. References

1. Cantagrel, G.; Meyer, C.; Cossy, J. *Synlett* **2007**, 2983-2986.

4. ^{1}H and ^{13}C NMR Spectra

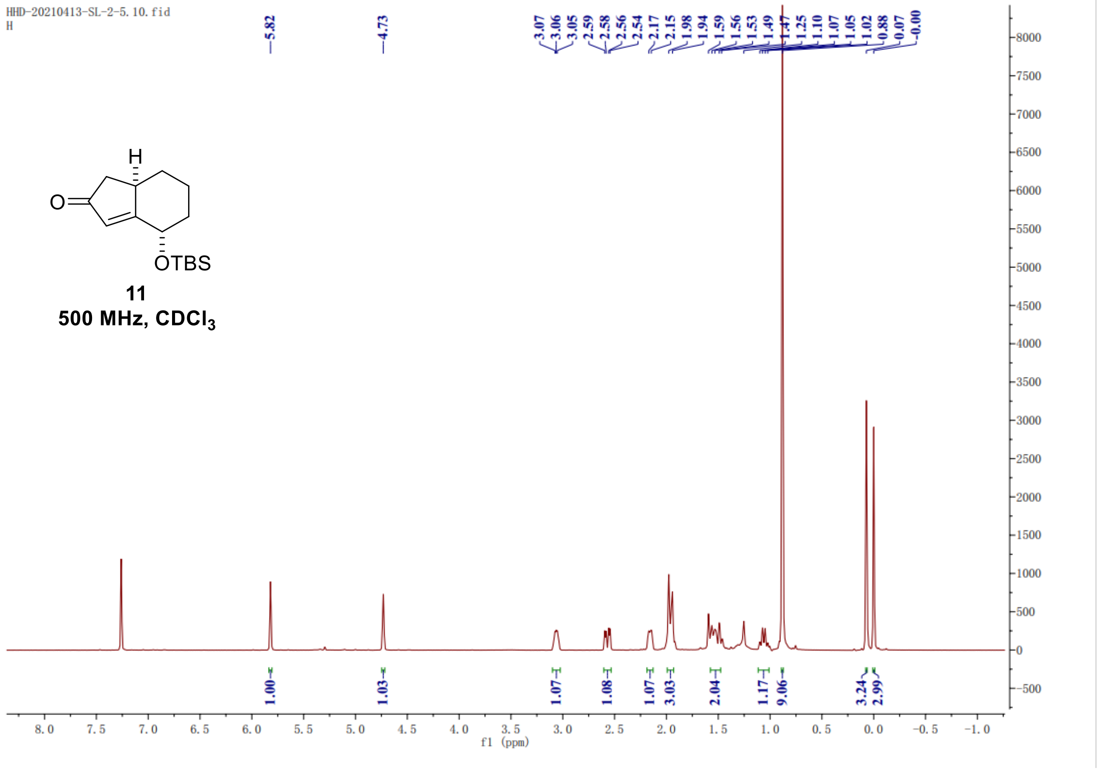

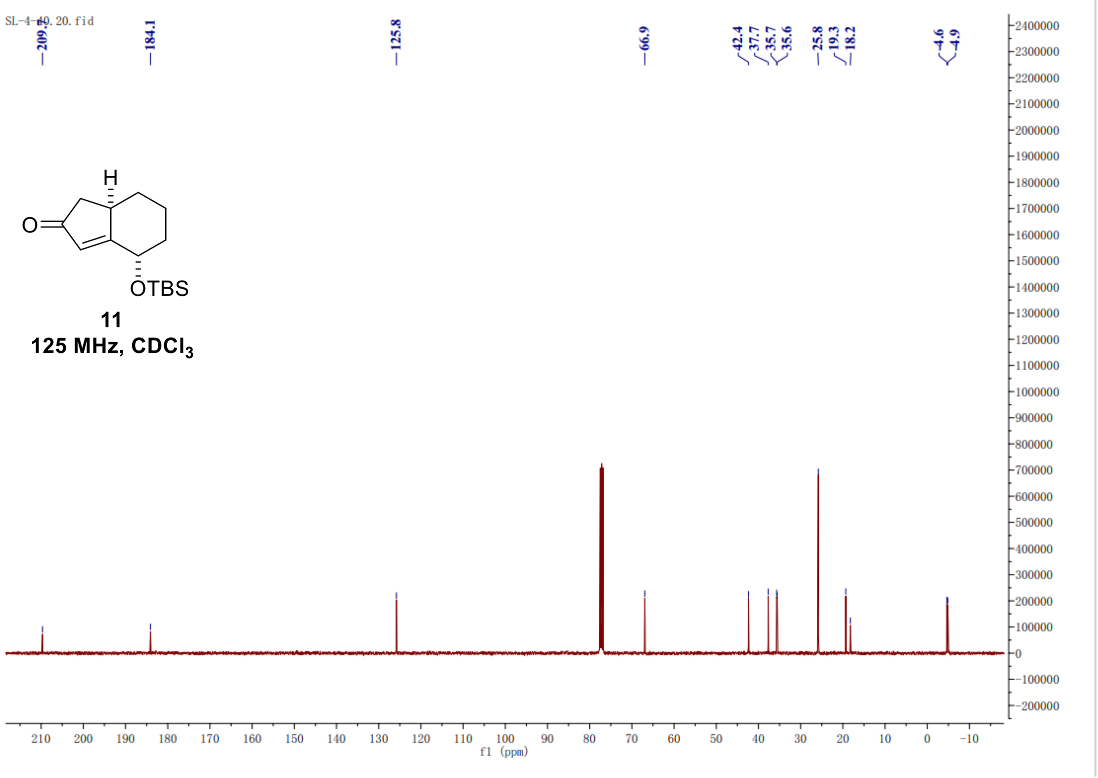

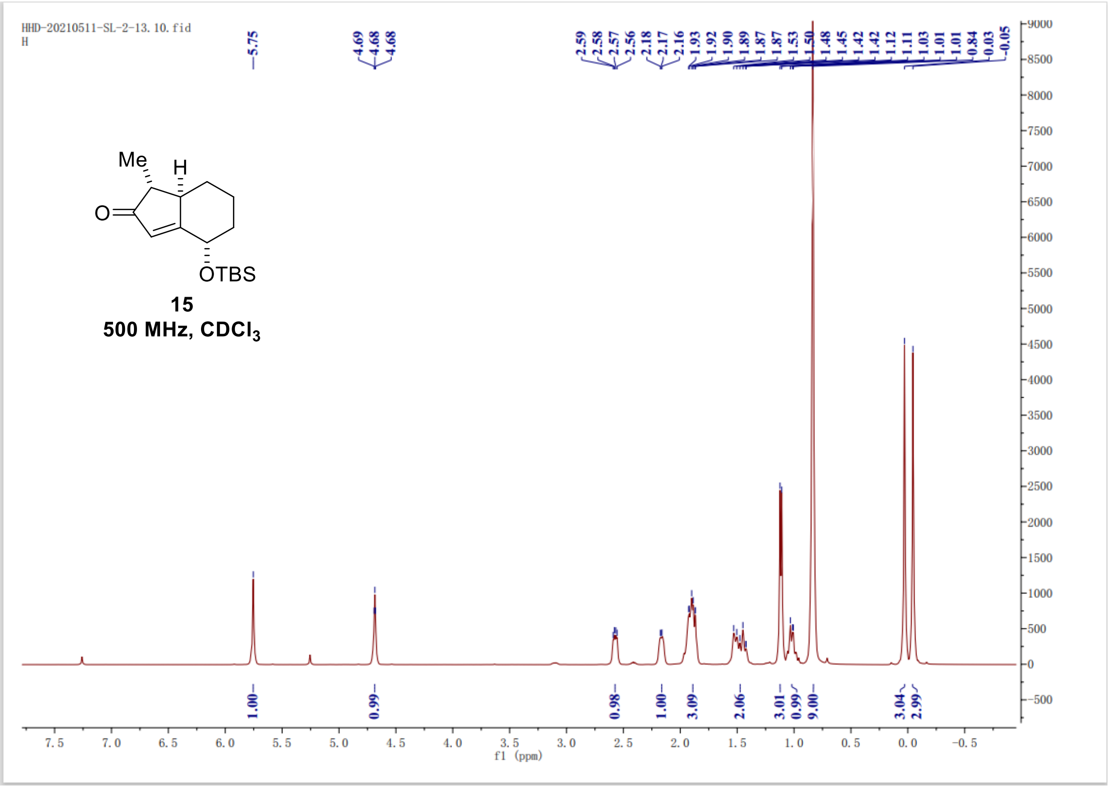
HHD-20210511-SL-2-13.10.fid
H
15
500 MHz, CDCl3
f1 (ppm)

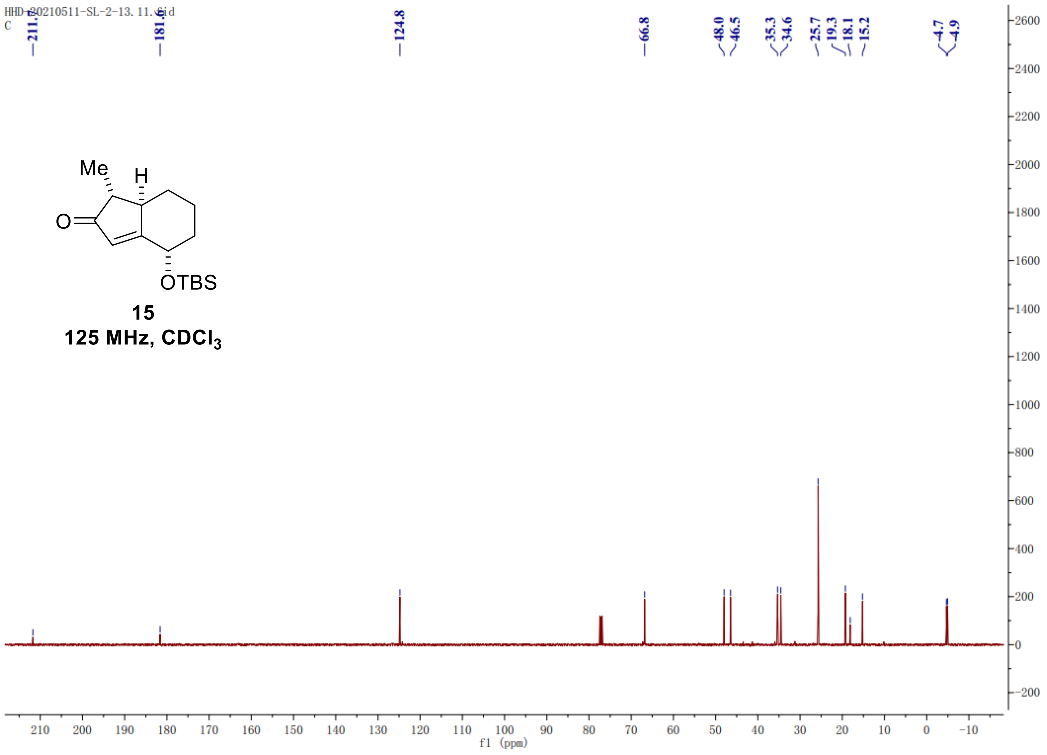
HHD-20210511-SL-2-13.11.fid
C
15
125 MHz, CDCl3
f1 (ppm)

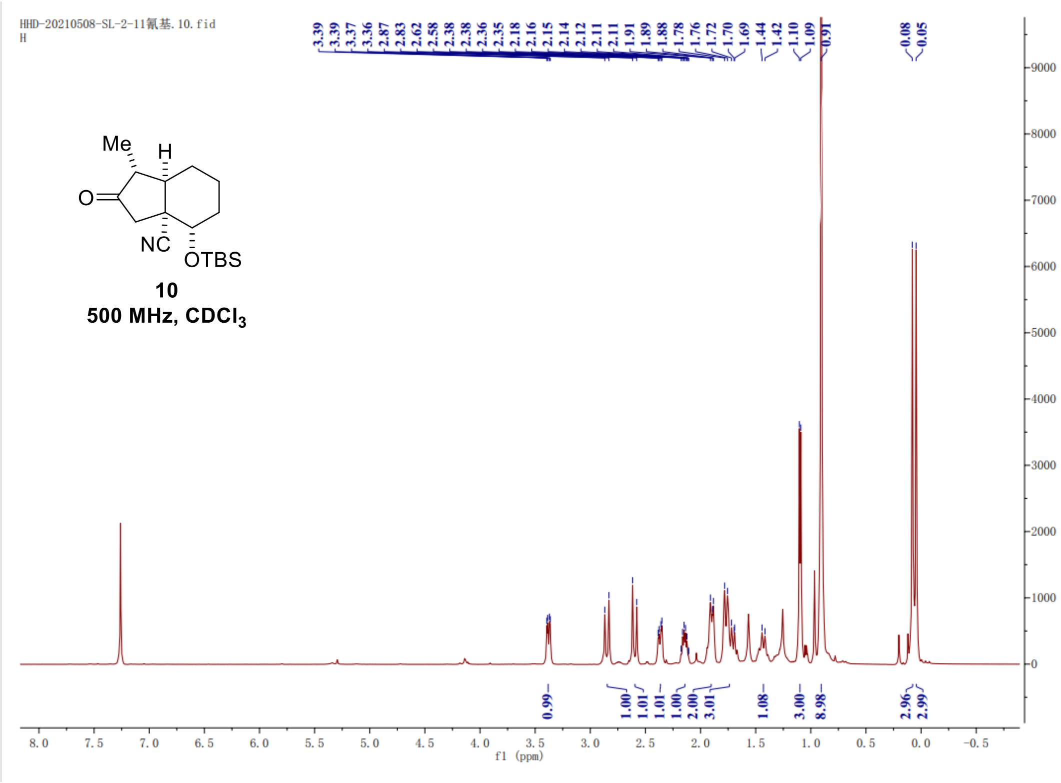
HHD-20210508-SL-2-11氰基.10.fid
H
3.39 3.39 3.37 3.36 2.87 2.83 2.62 2.58 2.38 2.38 2.36 2.35 2.18 2.16 2.15 2.14 2.12 2.11 2.11 1.91 1.89 1.88 1.78 1.76 1.72 1.70 1.69 1.44 1.42 1.10 1.09 0.91
0.08 0.05
Me
H
O
NC
OTBS
10
500 MHz, CDCl3
0.99 1.00 1.01 1.01 1.00 2.00 3.01 1.08 3.00 8.98 2.96 2.99
f1 (ppm)

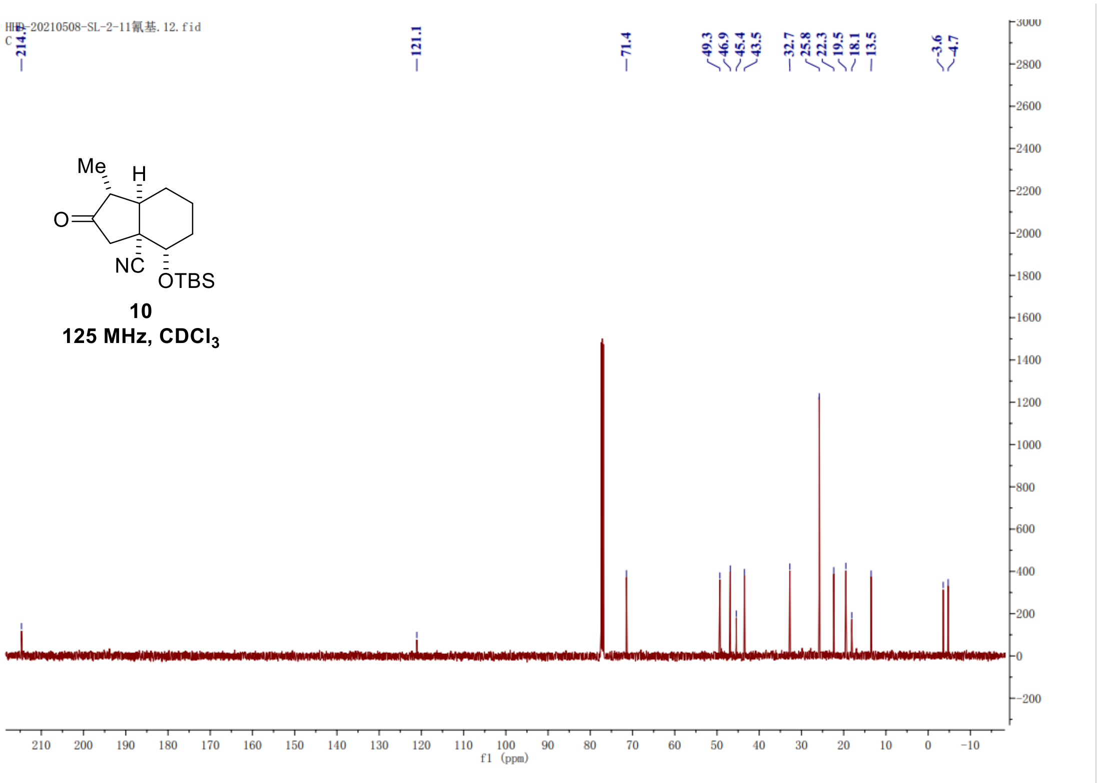
HHD-20210508-SL-2-11氰基.12.fid
C
214.
121.1
71.4
49.3 46.9 45.4 43.5
32.7 25.8 22.3 19.5 18.1 13.5
-3.6 -4.7
Me
H
O
NC
OTBS
10
125 MHz, CDCl3
f1 (ppm)

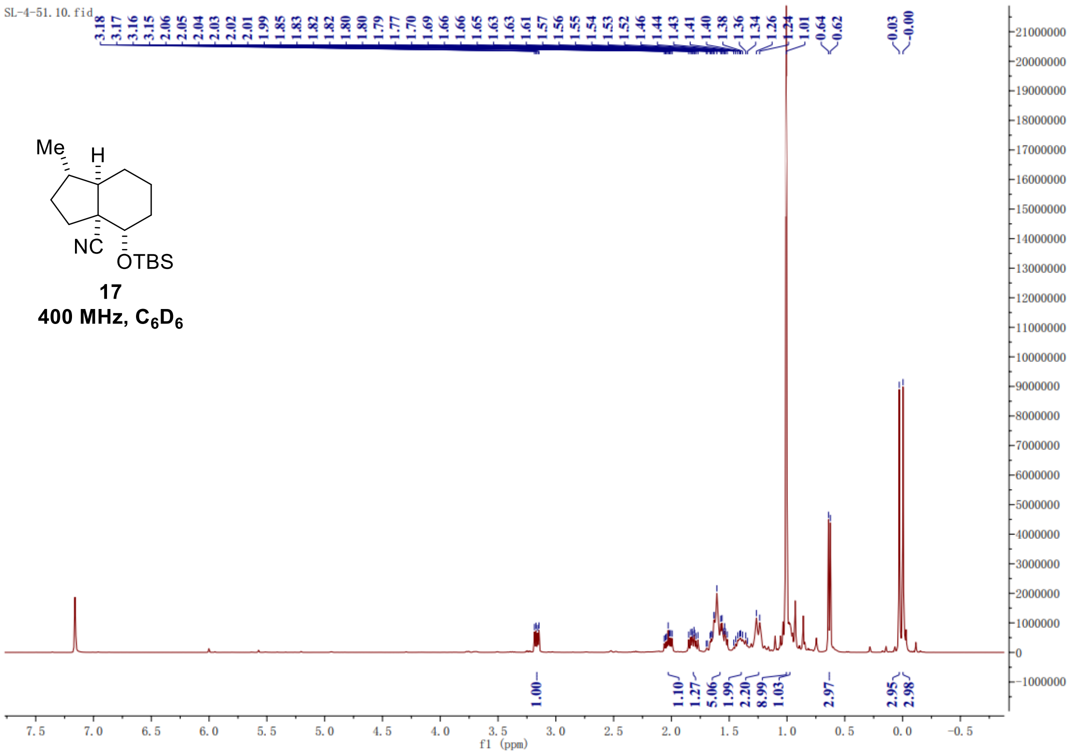
SL-4-51.10.fid
Me
H
NC
OTBS
17
400 MHz, C6D6
f1 (ppm)

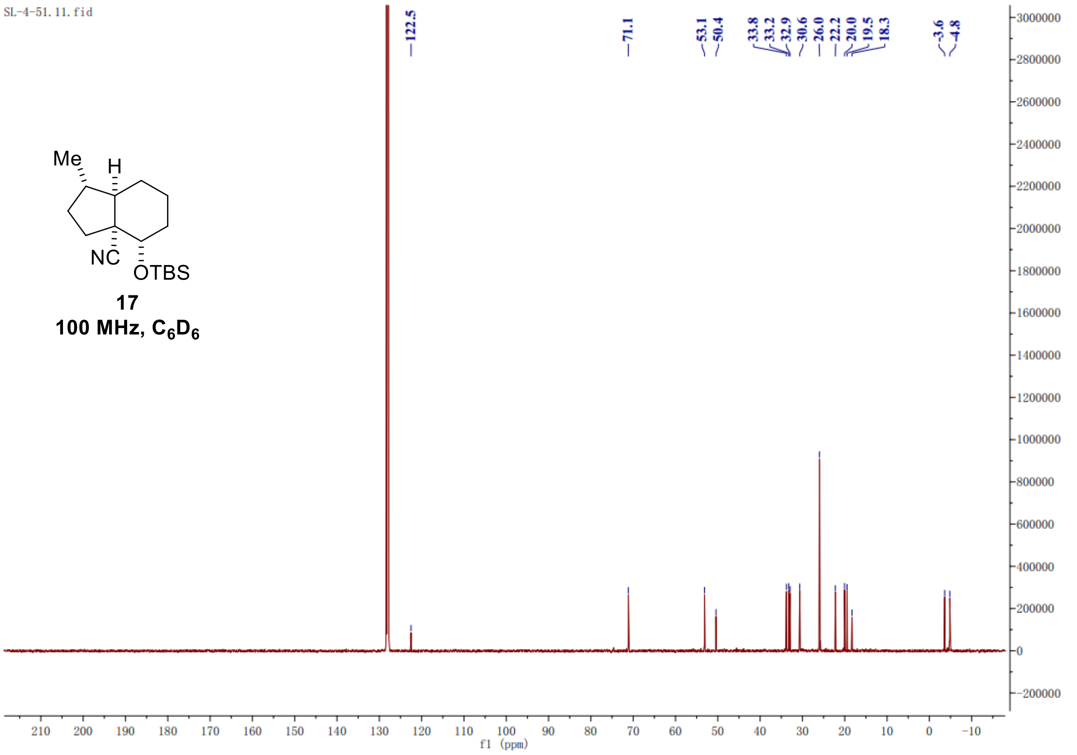
SL-4-51.11.fid
122.5
71.1
53.1
50.4
33.8
33.2
32.9
30.6
26.0
22.2
20.0
19.5
18.3
-3.6
-4.8
Me
H
NC
OTBS
17
100 MHz, C6D6
f1 (ppm)

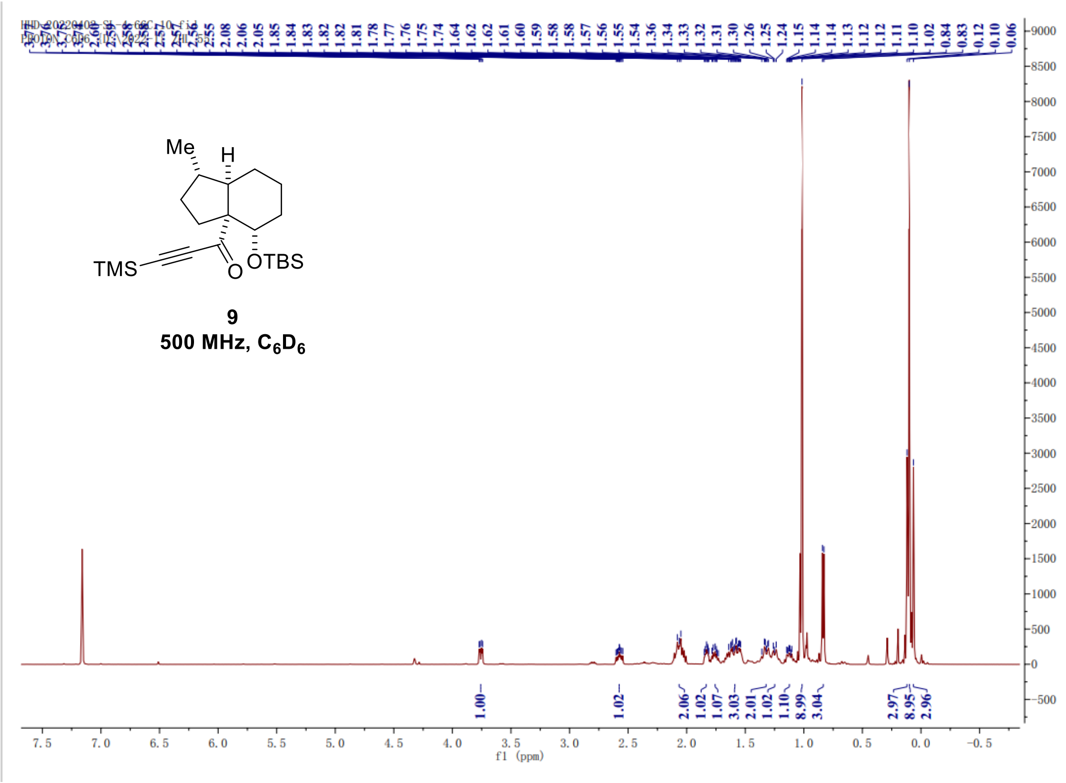
Me
H
TMS
O
OTBS
9
500 MHz, C6D6
1.00
1.02
2.06
1.02
1.07
3.03
2.01
1.02
1.10
8.99
3.04
2.97
8.95
2.96
f1 (ppm)

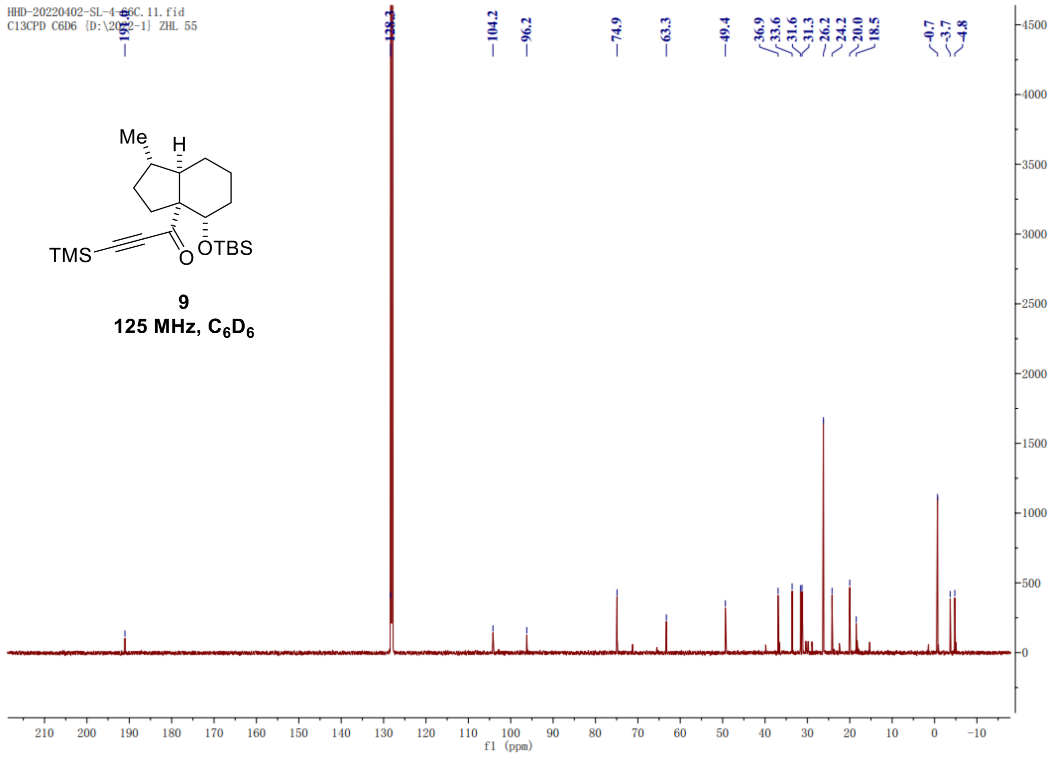
HHD-20220402-SL-4-66C.11.fid
C13CPD C6D6 {D:\2022-1} ZHL 55
128.3
104.2
96.2
74.9
63.3
49.4
36.9
33.6
31.6
31.3
26.2
24.2
20.0
18.5
-0.7
-3.7
-4.8
Me
H
TMS
O
OTBS
9
125 MHz, C6D6
f1 (ppm)

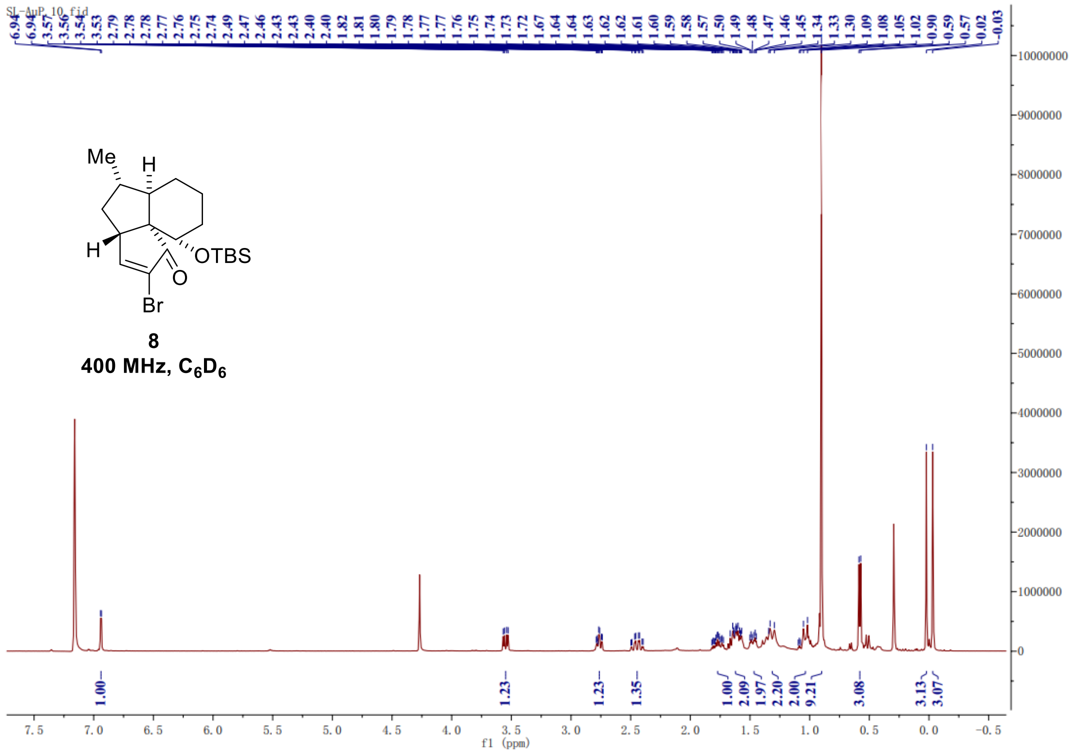
SL-AuP.10.fid
Me
H
H
OTBS
O
Br
8
400 MHz, C6D6
f1 (ppm)

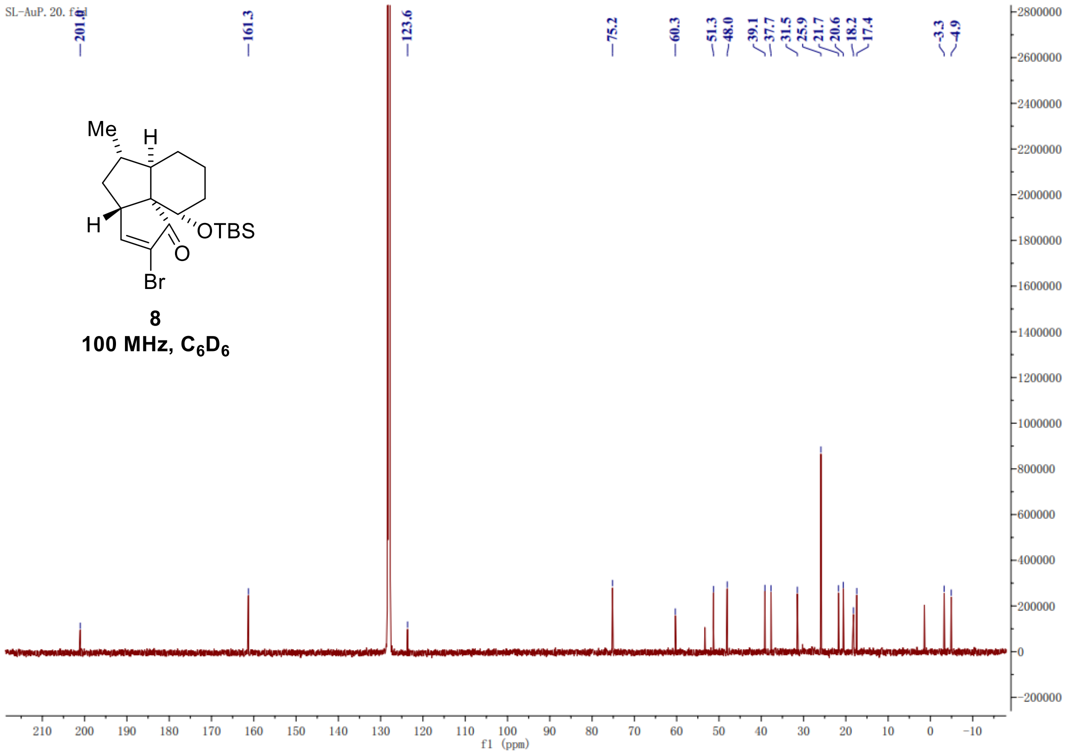
SL-AuP.20.fid
Me
H
H
OTBS
O
Br
8
100 MHz, C6D6
f1 (ppm)
